# How to Cure Rhus Poisoning

**Published:** 1893-10

**Authors:** C. H. Evans

**Affiliations:** Chicago, Ill.


### HOW TO CURE RHUS POISONING.

C. H. Evans, M. D., Chicago, Ill.

It is sincerely to be hoped that Homœopathy never will be credited as identical with the theory of it set forth in the paragraph quoted by Mr. Rabe in the August number at page 445. It is the caricature of Homœopathy that the allopathic school have set up, "Take a hair of the dog that bit you." Hahnemann never taught such doctrine, and not a line approaching it can be found in *The Organon*. It is isopathy, not Homœopathy. Webster defines these as follows: Isopathy—The cure of a disease by means of the virus of such a disease. The theory of curing a diseased organ by the use of the analogous organ of a healthy (diseased) animal. Homœopathy—The art of curing founded on resemblances; the theory and its practice that disease is cured by remedies which produce on a healthy person effects similar to the symptoms of the complaint under which the patient suffers.

So it is not the identical virus, but the *resemblance* to a disease that a drug has produced upon healthy persons that constitutes Homœopathy. The former is represented by Pasteur, Koch, Brown-Sequard, *et al.*, and the few who have adopted the nosodes, while the latter was taught by Hahnemann and is practiced to-day as the science of therapeutics.

The banner of isopathy has no place in the ranks of Homœopathy. To borrow another simile it is an exotic.

[What Dr. Evans says above is perfectly true. We think, though, that in quoting the paragraph referred to Mr. Rabe, who is a lawyer, meant merely to show how closely the old school approaches to Homœopathy without giving any credit to the law of the similars, even using the very medicines they are accustomed to denounce and ridicule. We all know that in treating Rhus-tox. poisoning we sometimes meet with cases that call so loudly for Rhus-tox. that we feel compelled to give it—in a high potency—and with curative effect. "A hair of the dog that bit you" is but a subterfuge to avoid giving the credit to the hated system of treatment.—ED.]

